# Pneumoblastome de l’adulte: rapport d’un nouveau cas et revue de la litterature

**DOI:** 10.11604/pamj.2017.28.198.13907

**Published:** 2017-11-02

**Authors:** Mustapha Azzakhmam, Fouad Zouaidia, Ahmed Jahidd, Zakia Bernoussi, Kaoutar Znati, Asmae Lakhdissi, Mohamed Bouchikh, Najat Mahassini

**Affiliations:** 1 Labaoratoire d'Anatomie Pathologique/Hopital Militaire Mohamed V, Rabat, Maroc; 2 Laboratoire d'Anatomie Pathologique/Centre Hospitalier Universitaire Avicenne, Rabat, Maroc; 3 Institut National d'Oncologie INO Moulay Abdellah, Rabat, Maroc

**Keywords:** Pneumoblastome biphasique, adulte, rare, Biphasic pneumoblastoma, adult, rare

## Abstract

Le blastome pulmonaire décrit surtout chez les enfants, est une tumeur rare. Elle représente 0.25 à 0.5% de toutes les tumeurs pulmonaires avec un pronostic très grave. Sur le plan histologique, il s'agit d'une tumeur à double composante: une composante épithéliale et une composante mésenchymateuse. Cliniquement, il s'annonce souvent par des douleurs thoraciques, de la toux, des hémoptysies et de la dyspnée, mais reste asymptomatique dans environ 40% des cas. Nous rapprtons le cas d'une femme de 25 ans, sans antécédents particuliers, qui se plaignait de dyspnée, de toux et de douleurs basithoraciques gauches. l'exploration radiologique avait montré une large masse basithoracique du poumon gauche. La biopsie a été réalisée et avait ramené uniquement du matériel nécrotique. La pièce de résection était largement nécrosée, le tissu viable examiné au miroscope avait révélé un pattern biphasique, composée de tissu épithélial malin, associé à un tissu mésenchymateux malin, caractéristiques du pneumoblastome biphasique. La patiente a bénéficié d'une chimiothérapie puis radiothérapie. Le contrôle a montré une récidive et la patiente a été mise sous deuxième ligne de chimiothérapie.

## Abstract (en)

Pulmonary blastoma is a rare tumor which has been mainly reported in children. It accounts for 0.25-0.5% of all pulmonary tumors, with a very serious prognosis. Histologically, it is a tumor composed of two components: an epithelial component and a mesenchymal component. Clinically, it usually manifests as chest pain, cough, hemoptysis and dyspnea, but it is asymptomatic in approximately 40% of cases. We report the case of a 25 year old woman, with no previous medical history, who complained of dyspnoea, cough and left basithoracic pain. Radiological evaluation showed large basithoracic mass in the left lung. A biopsy was performed which only showed necrotic material. The surgical specimen was largely necrotic. The viable tissue was examined with the miroscope which showed biphasic pattern composed of malignant epithelial tissue associated with malignant mesenchymal tissue, typical of biphasic pneumoblastoma. The patient underwent chemotherapy and radiation therapy. Follow-up examination showed a recurrence, thus the patient underwent second line chemotherapy.

## Introduction

Le pneumablastome est une tumeur maligne rare du poumon, il représente environ 0.25-0.5% de toutes les tumeurs pulmonaires [1-3]. Histologiquement, il ressemble au tissu fœtal et peut exprimer aussi bien les caractéristiques épithéliales et mésenchymateuses. Malgré ses origines embryologiques, cette tumeur affecte de façon prédominante l'adulte [4]. Très peu de cas, classés comme pneumablastome de l'adulte ou de l'enfant, sont rapportés dans la littérature. Chez l'adulte, la tumeur se présente souvent comme une large masse symptomatique a l'origine de toux, d'hémoptysies, de fièvre et de douleurs thoraciques.

## Patient et observation

**Figure 1:**
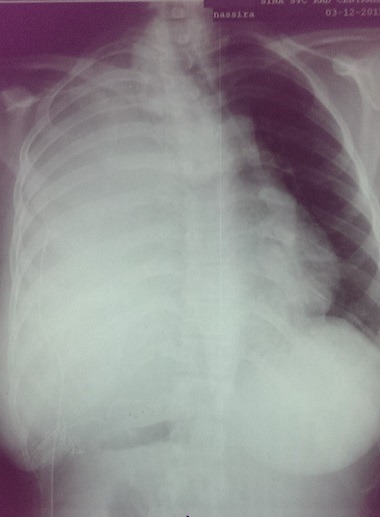
Comblement de l’hémithorax gauche par une masse tissulaire

**Figure 2:**
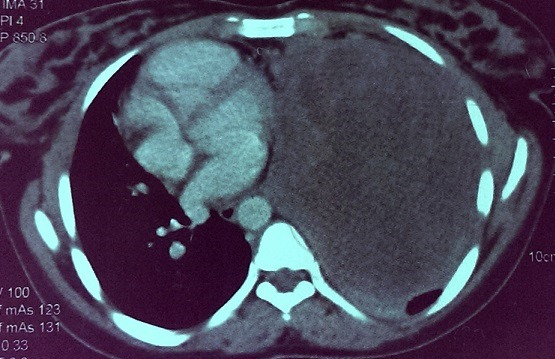
Masse hypodense de l’hémithorax gauche refoulant le médiastin à droite

**Figure 3:**
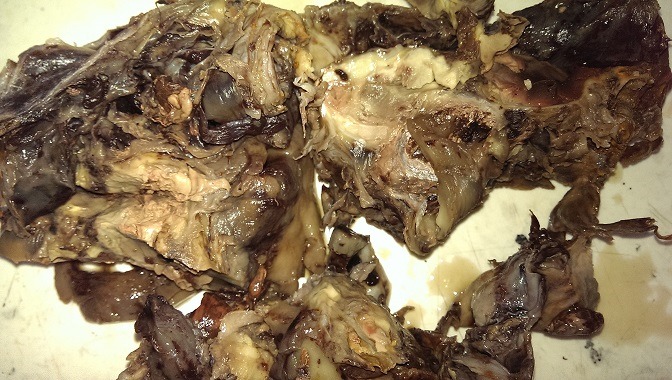
Aspect macroscopique du poumon gauche montrant de larges remaniements nécrotiques, kystiques et hémorragiques

Il s'agit d'une patiente de 25 ans qui a été admise au service de pneumologie de l'hôpital Avicenne de rabat pour des douleurs basithoraciques gauches irradiant vers l'épaule gauche et exacerbées lors de l'inspiration, associées à une dyspnée stade II de la NYHA, avec toux et crachats abondants. L'examen clinique de cette patiente avait retrouvé une diminution des vibrations vocales et du murmure vésiculaire, une sensibilité lors de la palpation de l hémithorax gauche et une déviation des bruits cardiaques vers le coté droit. Le tout évoluant dans un contexte d'apyrexie et d'amaigrissement non chiffré. Une radiographie pulmonaire a été réalisée et a montré un comblement de l'hémithorax gauche par une large masse de tonalité tissulaire, refoulant le cœur vers la droite (Figure 1). Une tomodensitométrie a été réalisée et a montré la présence d'une masse thoracique gauche mesurant 17 x 13cm, n'épargnant que la partie supérieure du lobe, hétérogène et hypodense, réhaussée en périphérie et refoulant le médiastin vers la droite avec atélectasie du lobe inférieur et un épanchement basithoracique gauche. Le scanner n'a pas objectivé de lésions nodulaires évolutives, ni d'adénomegalie mediastinale ou de masses pariétales (Figure 2). Une biopsie scannoguidée a été réalisée et n'a porté que sur du matériel nécrotique sans tissu vivace. La patiente a été prise en charge en chirurgie thoracique et a bénéficié d'une pneumectomie gauche. L'examen macroscopique, après fixation au formol, avait porté sur une pièce de pneumectomie gauche de 26cm de grand axe et dont la coupe montrait un aspect translucide avec des remaniements kystiques et hémorragiques. Elle était largement nécrotique. De multiples prélevements ont été effectués pour ramenr un maximum de tissu viable, et qui se localisait surtout en périphérie (Figure 3).

**Figure 4:**
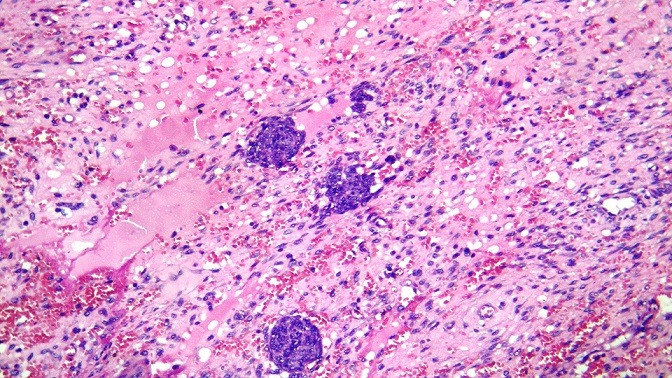
Morules épithéliales tumorales au sein d’un tissu mésenchymateux malin (HE, G x 20)

**Figure 5:**
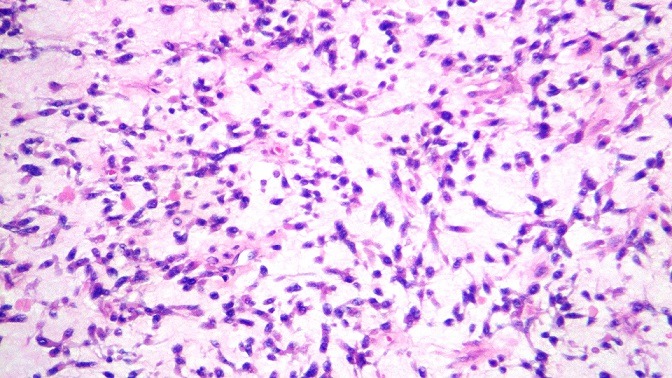
Tissu mésenchymateux malin (HE, G x 40)

**Figure 6:**
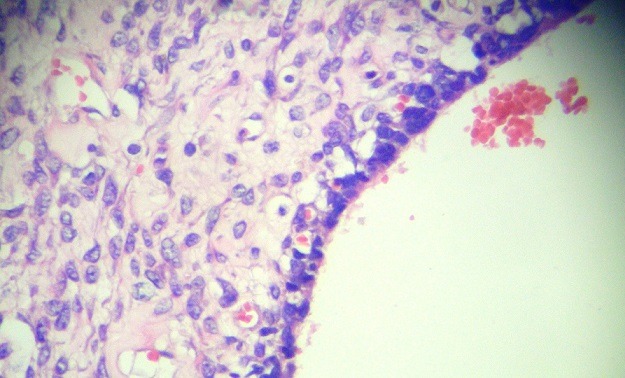
Zones kystiques bordées de cellules épithéliales malignes adjacentes aux cellules mésenchymateuses (HE, G x 40)

**Figure 7:**
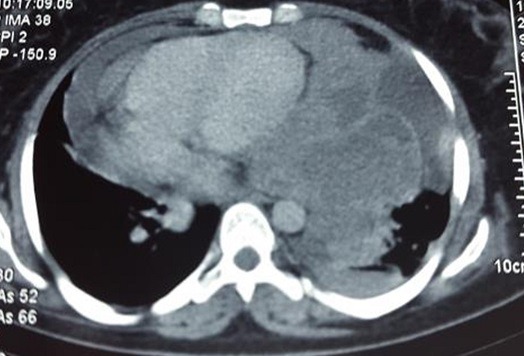
TDM thoracique montrant la recidive tumorale au niveau de l’hémithorax gauche

L'étude au microscope des nombreuses coupes réalisées, a montré une prolifération tumorale a double contingent; un contingent mésenchymateux a cellules fusiformes aux noyaux anisocaryotiques et hyperchromatophiles avec des limites cellulaires imprécises et de nombreuses figures de mitoses, et qui représente la majeure partie de cette lésion. Ce contingent comporte également des remaniements kystiques. A ce contingent, s'associe un contingent épithélial, fait de cellules épithéliales atypiques aux noyaux basophiles pseudo-stratifiés par endroits et au cytoplasme peu abondant, des morules de cellules épithéliales malignes sont fréquemment observées. Il n a pas été observé de composantes hétérologues. Les limites de résection chirurgicales étaient tumorales. L'analyse morphologique a permis de retenir le diagnostic de pneumablastome de type biphasique sans recours aux techniques d'immunohistochimie complémentaires (Figure 4, Figure 5, Figure 6). La TDM thoracoabdominale post opératoire de contrôle, et la scintigraphie osseuse n'ont pas montré de récidive locale ou de métastases a distance. La patiente a été readressée en service de chirurgie thoracique pour éventuelle ré-intervention car les marges de résection étaient tumorales. Vu la présence de certains facteurs de mauvais pronostic (taille, type biphasique, taille de la tumeur dépassant 5cm), une chimiothérapie adjuvante a base de d'Etoposide et de Cisplatine a été instauré, puis complétée par une radiothérapie à cause de la positivité des marges de résection. Le contrôle radiologique avait montré une récidive tumorale locale sous forme d'une masse hétérogène mesurant 15 x 112 x 9.7cm, localisée au niveau de l'hemithorax gauche avec hypodensité centrale comprimant le cœur et épanchement pleural et péricardique (Figure 7). La patiente a reçu une radiothérapie palliative puis a été mise sous une deuxième ligne de chimiothérapie.

## Discussion

Le blastome pulmonaire ou pleuropneumoblastome est une tumeur rare et agressive du poumon, elle représente environ 0.25-0.5% de toutes les tumeurs pulmonaires [5]. Cette tumeur ressemble morphologiquement au tissu pulmonaire fœtal avant l'âge de 4 mois [5]. Décrite la première fois par Bernard en 1952 [6], elle a été subdivisé par Koss et al en trois sous groupes: le blastome pulmonaire biphasique, le blastome monophasique avec expression épithéliale prédominante et enfin le pleuro-pneumoblastome a expression mésenchymateuse [1, 4, 7-9]. Le sous type monophasique a prédominance épithéliale, appelé également adénocarcinome fœtal, comporte des glandes tumorales associées à un tissu mésenchymateux d'allure bénigne, alors que le pleuropneumoblastome mésenchymateux comporte des glandes d'apparence bénigne. le pneumablastome biphasique classique comporte des glandes et du tissu mésenchymateux qui sont tous les deux embryonnaires et malins [5]. Historiquement, le terme de blastome pulmonaire avait inclu l'adenocarcinome fœtal pur, le pleuropneumoblastome et le pneumoblastome biphasique classique. En 1999 et en 2004, dans la nouvelle classification de l'OMS [5, 6, 10], l'adénocarcinome fœtal bien différencié et le pleuropneumoblastome ont été séparés des tumeurs biphasiques [6]. Le blastome pulmonaire biphasique est considéré actuellement comme un sous type des carcinomes sarcomatoides pulmonaires selon la nouvelle classification de l'OMS 2004 [5, 11]. Chez l'adulte, la tumeur se présente comme une large masse thoracique à l'origine de douleurs, de toux, de dyspnée. Cependant, 40% des patients peuvent être asymptomatiques [3]. Les difficultés diagnostiques, relèvent du pléomorphisme morphologique de cette tumeur; en effet la cytologie identifie fréquemment et seulement un adénocarcinome, c'est pourquoi le diagnostic doit être suspecté lorsqu'une aspiration à l'aiguille montre des populations séparées de cellules épithéliales et de cellules stromales [12].

Approximativement, 80% des pneumablastomes biphasiques sont retrouvés chez l'adulte [13] et avec deux pics d'âges de fréquence, le premier à la quatrième décade et quelques rares cas ont été rapportés à l'âge de 80 ans. Il existe une légère prédominance chez les fumeurs [14]. L'examen clinique peut révéler une réduction des vibrations vocales et du murmure vésiculaire. Les bilans biologiques sont non spécifiques. Le pneumoblastome se présente presque toujours comme une large masse unilatérale bien circonscrite, et solitaire sur la radiographie pulmonaire standard. Vu la localisation périphérique, le plus souvent, le diagnostic est obtenu par bronchoscopie dans 25% des cas [15]. Mais le scanner reste l'examen radiologique de choix. L'intérêt du TEP scan reste peu connu dans le staging radiologique de cette tumeur [5]. Les diagnostics différentiels radiologiques comprennent aussi bien des lésions bénignes, comme l'hamartome, le chondrome, le fibrome pleural, et l'hamartome sclérosant, ainsi que des lésions malignes comme les autres tumeurs primitives ou secondaires du poumon [5]. Les blastomes pulmonaires sont des tumeurs biphasiques appartenant au groupe des carcinomes sarcomatoides [10], comprenant aussi les carcinosarcomes (définis comme étant des tumeurs malignes ayant une mixture de carcinomes et de sarcomes contenant des éléments hétérologues comme le cartilage, l'os, ou le muscle squelettique) et les carcinomes pléomorphes (idem mais sans composante hétérologue). Le contingent épithélial des blastomes biphasiques est composé de tubes bordés de cellules épithéliales riches en glycogène et non ciliées, et qui ressemblent au poumon fœtal au stade de développement. L'apparence embryonnaire du stroma est due à la petite taille, aux formes ovalaires et fusiformes des cellules, et à la matrice myxoide. Classiquement, le stroma n'exprime pas les cytokératine ni les marqueurs pulmonaires [16].

L'étude immunohistochimique demeure trés utile dans le diagnostic des pneumoblastomes biphasiques, particulierement, la combinaison de marqueurs épitheliaux comme l'anitgéne membranaire epithelial (EMA), les cytokératines (CAM5.2, CK5/6,CK AE1/AE3), et les marqueurs mésenchymateux comme la Vimentine; chaque composant tissulaire va être plus prononcé par rapport à l'autre. CD34 et la protéine S-100 marquent les deux composantes, épithéliale et mesenchymateuse [16]. De même, Hansen et al ont rapporté comme nouveau marqueur des blastomes pulmonaires biphasiques, l'expression du KIT (CD6117) par cette tumeur [16]. La Béta-caténine pourrait jouer un rôle dans la tumorogénèse des blastomes pulmonaires classiques: ses localisations atypiques nucléaires/cytoplasmiques, mises en évidence par marquage immunohistochimique, ont été raportées utiles pour distinguer le blastome classique d'une variante blastomatoide du carcinosarcome et de l'adénocarcinome de type fœtal de haut grade [16]. Les pneumoblastomes de type II et III devront être differenciés des tumeurs suivantes: le mésothelium sarcomatoide; qui ne comporte souvent pas de petites cellules malignes, et dont le profil immunohistochimique se caractérise par un marquage positif par l'actine et la desmine. Le diagnostic du mésothéliome sarcomatoide peut être retenu si les deux anticorps anti-calrétinine et anti-cellules mésothéliales sont positifs. Le rhabdomyosarcome embryonnaire est à prendre en considération. En effet, une différenciation rhabdomyoblastique des cellules peut se voir. Le siège de survenue des deux entités est trés différent: cavité nasale et vagin pour le rhabdomyosarcome et poumon pour le pneumoblastome. En plus, le rhabdomyosarcome est toujours positif pour les marqueurs myogéniques. Le synovialosarcome peut être facilement distingué vu l'âge de survenue et le siége. Les cellules fusiformes y sont de morpholgie differente des celles du pneumoblastome, et se disposent au debut prés des vaisseaux, puis de facon désordonnée tardivement. Il a une différenciation bidirectionnelle, mais sans différenciation des cellules mésenchymateuses primitives, ni des cellules rabdomyoblastiques. Le marquage immuno-histochimique peut être simultanément positif pour la CK, l'EMA, et la vimentine [16]. Les tumeurs neuro-éctodermiques primitives (PNET), se caractérisent par la présence des rosettes de Homer-Wright, des tubes épendymaires et les rosettes de Flexner Winsteiner. L'immuno-histochimie indiquera une différenciation neuroide [16]. L'excision chirurgicale reste le traitement de choix [6, 10]. Le pronostic rapporté dans la littérature est généralement pauvre, avec une survie limitée a 2 ou 3 ans après diagnostic.

Les pneumoblastomes de type II et de type III, sont des tumeurs trés agressives; malgrés les différentes modalités thérapeutiques, le taux de survie est de l'ordre de 62% à 2 ans, et de 42% à 5 ans [17-19]. L'étude d'une série de 350 cas menée par Messinger et al [20], avait confirmé que le type histologique du pneumoblastome reste le facteur pronostique le plus puissant: le resultat était meilleur pour le type I, contrairement aux types II et III. Les résultats pour le type II restaient significativement meilleurs par rapport au type III. Cette série a conclu que la forme épithéliale (typeI), aurait le meilleur pronostic. Les types II (biphasique) et III sont les formes agressives, avec haut risque de récidive et taux important de décès. Le type histologique et la présence de metastase au moment diagnostic, seraient les plus importants facteurs pronostiques rattachés au traitement. La différence en survie moyenne dépend de l'atteinte des ganglions médiastinaux. Cette dernière décade, un meilleur pronostic a été observé lorsqu'une chimiothérapie néo-adjuvante pré-opératoire est administrée [13].

## Conclusion

Le pneumoblastome de l'adulte est une tumeur pulmonaire inhabituelle qui se présente comme une masse invasive. Le diagnostic, le traitement et le suivi devront relever d'une équipe multidisciplinaire. Une stratégie basée sur une chirurgie radicale et une radio-chimiothérapie peut être le meilleur choix pour un traitement éfficace.

## Conflits d’intérêts

Les auteurs ne déclarent aucun conflit d'intérêts.
